# Antibacterial and Moisture Transferring Properties of Functionally Integrated Knitted Firefighting Fabrics

**DOI:** 10.3390/polym17212915

**Published:** 2025-10-31

**Authors:** Zhilin Teng, Zhen Li, Yue Zhang, Chentian Zhang, Liming Wang, Xinxin Li, Xing Jin, Rongwu Wang

**Affiliations:** 1 College of Textiles, Donghua University, Shanghai 201620, Chinalixinxin@dhu.edu.cn (X.L.); 2 Tianjin Fire Science and Technology Research Institute of MEM, Tianjin 300381, China

**Keywords:** micro and nano fibers, cross-scale interface, antibacterial and moisture-transporting, fire-retardant fabric

## Abstract

This research highlights the issue that large amount of sweat generated by metabolism cannot be discharged from the internal environment of traditional fire suits when firefighters are intensively operating in high-temperature environments. This is highly prone to bacterial growth, which brings much harm to their health. Therefore, this study aims to present a new fire-retardant fabric with both antibacterial and high hygroscopic properties. Blended fibers were used including aramid 1313 fibers with excellent flame retardancy and flame-retardant viscose fibers. By uniformly embedding antibacterial nanofibers into the microfiber aggregates and controlling the adhesion behavior at the cross-scale interfaces of micro–nano fibers, the fire-retardant yarns were endowed with both antibacterial and moisture-transporting properties. The bacterial inhibition rate was calculated by comparing colonies cultured on EF fabric versus NF fabric. Additionally, the antibacterial and moisture-wicking properties of the fabrics were verified through tests such as placing the fabrics vertically in liquid to measure the height of absorbed moisture. This prepared functionally integrated fabric has excellent antibacterial properties even after 50 washing cycles. Its antibacterial rate against *Escherichia coli* and *Staphylococcus aureus* kept a preferred result of 99%. Its moisture-transporting performance has also been significantly improved. Based on the above, this study has not only successfully developed a flame-retardant fabric with high antibacterial and moisture-wicking properties, but more importantly, the method demonstrates a degree of universal applicability.

## 1. Introduction

With the increasing demand for health protection and comfort, new fabrics with both antibacterial and moisture-conducting functions have become a research hotspot in the field of textiles [1,2,3,4,5]. Textiles endowed with antimicrobial and moisture-conducting functions are often studied from two aspects: yarn-based and fabric-based. One is through the yarn realizing a certain function, so that the fabric prepared by it realizes a certain function [6,7,8,9,10]. Yarn serves as the fundamental unit of fabric construction. Numerous studies have demonstrated that by modifying yarn properties, fabrics can be engineered to exhibit specific functionalities such as abrasion resistance, color fastness, enhanced strength, and softness [11,12,13,14,15]. Some scientists, through the use of denatured acrylic and bamboo yarn, made natural flame-retardant and antibacterial fabrics; the fabric combines comfort with natural antibacterial, flame-retardant, and other functions, not only for firefighting apparel fabrics to provide choices but also for children’s antibacterial and flame-retardant fabrics to provide an optimal program [16]. Some scientists have constructed fine capillary channels using controlled spinning techniques for nano-core yarns, thereby accelerating moisture conduction and endowing textiles with outstanding moisture-wicking properties [17]. However, the functions that can be realized only by relying on yarn is relatively singular; therefore, many scientists are committed to making the fabric realize certain functions by modifying the fabric, such as through fabric softness, antimicrobial, hydrophilic, and hydrophobic treatments [18,19,20,21,22]. To impart antibacterial properties to textiles, scientists prepared a Tencel/LMPET-TPU/triclosan composite membrane via needle-punching and lamination processes, endowing it with excellent waterproofing (100 kPa), windproofing (<0.1 cm^3^/cm^2^/s), and antimicrobial characteristics. This composite membrane utilizes triclosan to disrupt bacterial cell membranes, effectively inhibiting the growth of *E. coli* and *S. aureus* [23]. Qin et al. prepared antimicrobial flame-retardant cotton fabrics by compounding ammonium diphosphate (AP) as a flame-retardant and cross-linking agent as well as a one-pot method using hydroxyl-containing N-halamine precursors as antimicrobial agents with cotton fabrics [24]. Ma et al. employed wet spinning technology combined with impregnation to hydrophobically modify calcium alginate fibers using fluorocarbon resin, simultaneously enhancing their flame-retardant properties. This successfully transformed the natural polysaccharide fibers from hydrophilic to hydrophobic. By leveraging a wettability gradient, the hydrophobic-modified fluorocarbon resin calcium alginate fibers were combined with unmodified calcium alginate fibers to fabricate multifunctional protective textiles featuring a Janus-like structure with unidirectional water-repellency and flame-retardant properties [25]. Li et al. used phosphoric acid, modified chitosan and urea to successfully prepare flame retardants for the construction of antimicrobial and flame-retardant coatings on polyester/cotton fabrics through a protonation reaction, and the results showed that the fabrics possessed excellent antimicrobial and flame-retardant properties [26,27,28,29,30]. However, durability issues limit the application of the products in these research articles, and coatings on fabric surfaces can cause loss during use and washing. Therefore, many scientists have also conducted many non-coated functional studies. Sun et al. prepared fabrics with excellent moisture-conducting properties via poly(triethylene terephthalate)/poly(ethylene terephthalate) bicomponent filaments, which are typically characterized by cross-sectional molding that facilitates moisture transfer [31]. Many scientists have conducted in-depth research on antimicrobial and moisture conductive fabrics, but few scientists have researched multiple functions integrated into one fabric, which is mainly due to the coating on the fabric structure that confers good moisture conductivity and when destroyed, reduces moisture conductive multifunctional compatibility [32].

In this study, an integrated composite yarn composed of nanofibers and conventional fibers was developed, featuring both antimicrobial and moisture-wicking properties. Triclosan (TR) was incorporated into the precursor of the nanofiber spinning solution to impart antimicrobial functionality to the nanofibers. These nanofibers are distributed in a mosaic pattern throughout the yarn structure, enabling the entire yarn to exhibit antimicrobial effects. Importantly, the nanofibers are not only present on the yarn surface but also penetrate and are embedded within the porous structure of the traditional short-fiber web, forming a three-dimensional antibacterial network. This structure provides the synergistic inhibition of bacteria both on the surface and inside the yarn. Additionally, the distribution of nanofibers within the yarn creates finer capillary channels between the fibers, resulting in a higher rate of moisture conduction. This preparation method uses conventional fibers to effectively protect the nanofibers from damage during use, ensuring functional durability. When this functionally integrated yarn is used to produce knitted fabrics, the fabrics also acquire antimicrobial and moisture-wicking properties. This approach not only ensures long-lasting functionality but also significantly reduces production costs.

## 2. Methods and Characterizations

### 2.1. Materials

The conventional staple fibers used in this experiment were aramid 1313 fibers (Jiangsu Aoshen New Material Co., Ltd., Lianyungang, China), with a fineness of 1.5 denier/meter, length of 51 mm, breaking strength of 4.09 cN/denier, elongation at break of 32%, and moisture regain of 6.4%, and flame-retardant viscose fibers, with a fineness of 1.5 denier/meter, length of 40 mm, breaking strength of 2.58 cN/denier, elongation at break 15.09%, and moisture gain of 10.4% (Jiangsu Aoshen New Material Co., Ltd.). The polymer used for preparing antibacterial nanofibers in this study was polyacrylonitrile (PAN, Mw = 85,000, Shanghai McLean Biological Co., Ltd., Shanghai, China), and triclosan antimicrobial agent (TR, Shanghai Tihihi Ai Kasei Industrial Development Co., Ltd., Shanghai, China). The PAN and TR were dissolved in N,N-dimethylformamide (DMF, analytically pure, Shanghai McLean Biological Co., Ltd.) to obtain an antibacterial nanofiber spinning solution. The materials used for the antibacterial test include *Escherichia coli* (Shanghai Luwai Technology Co., Ltd., Shanghai, China), *Staphylococcus aureus* (Shanghai Luwai Technology Co., Ltd.), PBS buffer (Shanghai Doyou Biotechnology Co., Ltd., Shanghai, China), and nutrient agar (Sinopharm Chemical Reagents Co., Ltd., Shanghai, China), among others.

### 2.2. Preparation of Composite Yarns and Fabrics

**Figure 1:**
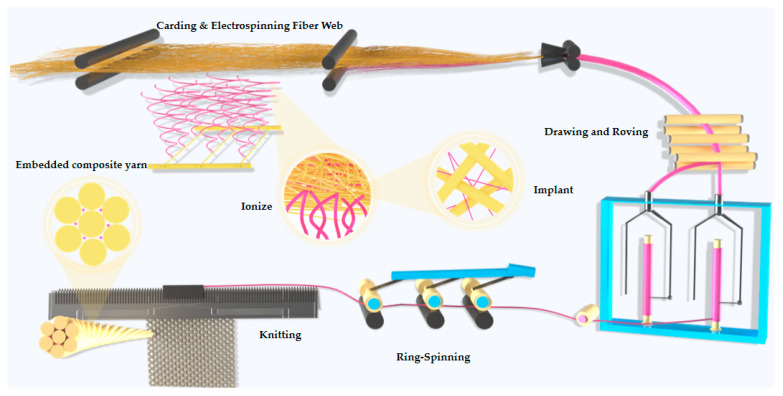
Function-integrated embedded composite yarn preparation process.

Figure 1 shows the process flow for preparing cross-scale embedded functional yarn (EY). First, 10 g of polyacrylonitrile (PAN) and 10 g of triclosan antibacterial agent were added separately to 90 g of DMF solution, stirred until dissolved, and an electrospinning solution was obtained. The conventional short fibers were then combed into a fiber web using a combing machine, and the loose fibers were subjected to combing treatment. A nanofiber macro-processing module was added below the fiber web to spray nanofibers containing antimicrobial agents onto the fiber web. In this way, the traditional fibers and antimicrobial nanofibers were firmly composite before the strip aggregation, and ultimately entered the triangular region formed by the strip aggregation to produce the mixed fiber strip. The blended fiber strand then entered the post-processing stage, which includes processes such as merging and short staple spinning. Under the action of roller stretching, controlled fractures occurred at the interfaces between fibers of different scales, enabling uniform dispersion of nanofibers and the formation of interfacial adhesion, thereby producing EY.

### 2.3. Characterization of Yarn and Fabric Properties

All yarns and fabrics were placed under standard temperature and humidity conditions for moisture absorption equilibrium for 24 h prior to testing. An electronic single yarn tester (YG061FQ, Laizhou Eeectron Instrument Co., Ltd., Laizhou, China) was used to test the breaking force of the yarns; the yarn hairiness tester (YG172A type, Shaanxi Changling Textile Electromechanical Technology Co., Ltd., Baoji, China) was used to test the yarn’s 3 mm hairiness index; fabric breaking strength was measured using an electronic fabric strength machine (YG026, Laizhou Eeectron Instrument Co., Ltd.). The bending stiffness of the fabric was measured using an electronic stiffness tester (LLY-01, Laizhou Eeectron Instrument Co., Ltd.) to characterize its softness, the breathability of the fabric was measured using a fabric breathability tester (YG461H, Shanghai Precision Instrument Co., Ltd., Shanghai, China), and the fastness to nanofibers in EF fabrics was tested by a fabric pilling tester (YG(B)502, Wenzhou Jigao Testing Instrument Co., Ltd., Wenzhou, China). The vertical capillary height, moisture content, and moisture evaporation rate characteristics of the fabric were determined according to the method specified in GB/T 21655.1-2023 [33,34]. The vertical capillary height test was conducted using a vertical capillary tester (Shenzhen Ruifeng Instrument Co., Ltd., Shenzhen, China), while the remaining tests were measured using a balance. The moisture diffusion area of the fabric was measured by pipetting 0.2 mL of water, with the tip of the gun tilted at 45° to place the water on the fabric to observe the diffusion area of the water at midday. The evaluation of the antimicrobial performance of the fabrics was tested by the oscillatory contact method combined with colony counting, and *E*. *coli* and *S*. *aureus* were selected as typical test strains for the study. After the experimental strains were activated by standard culture conditions, the fabric samples to be tested were cut into standard-sized samples and mixed with PBS buffer and a standardized bacterial suspension liquid system in a sterilized container. The mixed system was then transferred to a constant temperature oscillation culture device for contact culture at a certain temperature and a certain oscillation speed. After the contact culture was completed, the culture solution was appropriately diluted by the gradient dilution method, and each dilution of the bacterial solution was uniformly inoculated on the surface of the nutrient agar medium by the coating method. After a period of incubation, the proliferation inhibition effect of the fabric samples on the target strains was systematically analyzed by counting the colony forming units formed on the surface of the agar plates [35].

## 3. Results and Discussion

### 3.1. Basic Yarn and Fabric Performance Tests

**Figure 2:**
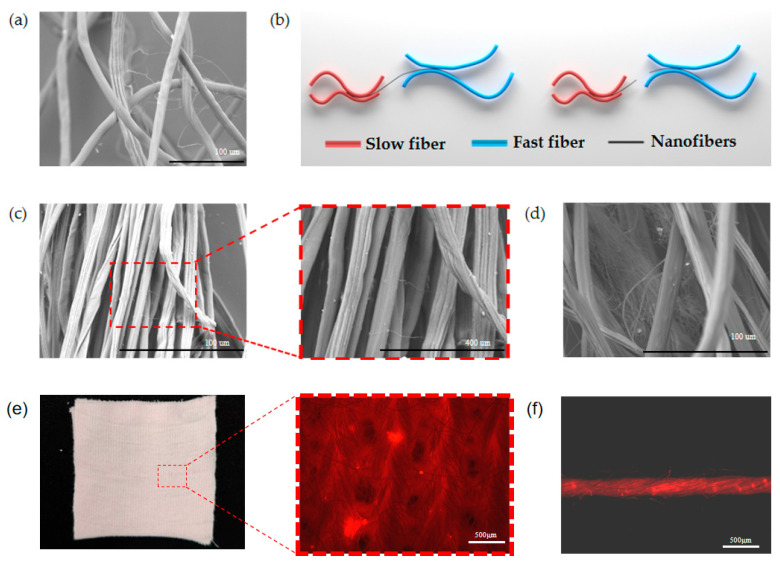
SEM micrographs of nanofibers and conventional fibers: (**a**) SEM image of composite fiber mesh of nanofibers and conventional staple fibers; (**b**) schematic diagram of nanofibers and conventional staple fibers adhesion movement; (**c**) SEM image of nanofibers adhesion fracture distribution in EY; (**d**) SEM image of nanofibers aggregation in EY; (**e**) clear images of EF fabric and fluorescence electron microscope images; (**f**) fluorescence electron microscope images of EF yarn.

As shown in Figure 2a, the morphology of nanofibers in the conventional staple fiber web presents a disordered state and forms an effective bond with the conventional fibers. The movement and transfer of conventional staple fibers is achieved by the speed difference between the front and back rollers to separate fast fibers, floating fibers, and slow fibers. Therefore, the breakage of nanofibers needs to have two conditions: (1) during the drafting process, the submicron fibers need to be held at one end and the other end subjected to tension; (2) the tension applied is not less than its own absolute breaking strength. As shown in Figure 2b, the difference between the adhesion force of fast fibers to nanofibers and that of slow fibers to nanofibers is greater than the breaking strength of nanofibers; the nanofibers break and move with the conventional fiber transfer to various locations in the yarn presenting a fully distributed state, and the nanofibers are subjected to the Van der Waals adhesion force of the fast fibers or the slow fibers in the actual composite fiber assemblage as calculated by Equation (1) [36], where *n* is the number of roots of fast or slow fibers contacted by nanofibers, *F_adh_* is the cross-scale fiber cross-section adhesion force, *A* is Hamaker’s constant, *L* is the effective interfacial contact length between the cross-scale fibers, *R*_1_ is the radius of nanofibers and *D* is the effective spacing between fibers.

(1) $$F_{adh}=F_{vdw}=\sum_{i=1}^{n}\frac{AL\left(i\right)}{8\sqrt{2}D^{5/2}}{\left(R_{1}\right)}^{1/2}$$

Figure 2c also shows that the nanofibers in the composite yarn are broken by stretching and move with the conventional staple fibers to various places in the yarn. Figure 2d shows that there is a state of nanofiber agglomeration in the yarn, and when the nanofibers are agglomerated, the conventional staple fibers do not have a significant force on them, which prevents them from transferring to the nanofiber twist. To more clearly observe the distribution state of nanofibers within fabrics and yarns, this study incorporated the fluorescent dye Rhodamine B into the electrospinning solution. The morphology of nanofibers within the fabric and yarn structure was then examined under a fluorescence microscope. Figure 2e,f present fluorescence electron micrographs of the fabric and yarn, respectively, alongside a clear image of the fabric. The clear fabric image reveals a pale pink hue, attributable to the fluorescent dye Rhodamine B imparting a pale pink color to the nanofibers. The presence of these nanofibers confers the pale pink appearance on the yarn. In the fluorescence electron micrographs, the red regions indicate nanofibers within the fabric and yarn. The brightened areas highlight the agglomeration of nanofibers within the yarn. Along the yarn’s axial direction, nanofibers exhibit relatively uniform distributions, with occasional instances of clustering.

**Figure 3:**
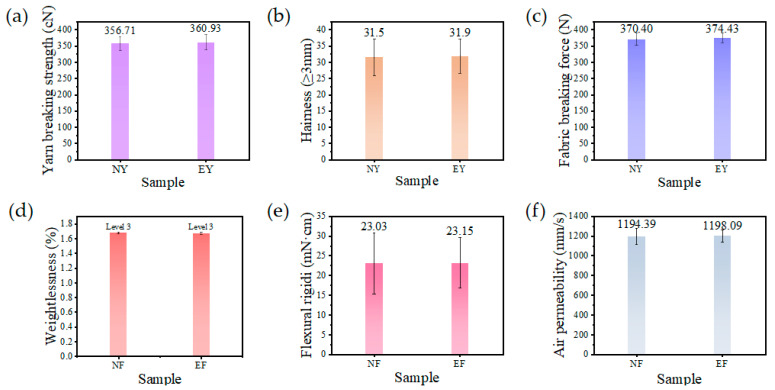
Basic yarn and fabric properties: (**a**) yarn breaking strength; (**b**) yarn hairiness index; (**c**) fabric breaking strength; (**d**) fabric pilling performance; (**e**) fabric bending stiffness; (**f**) fabric air permeability.

As shown in Figure 3a, the tensile strength of conventional yarn is 356.71 cN, while that of the embedded composite yarn is 360.93 cN. There is little difference in the tensile strength between the two yarns, as the nanofibers do not participate in the main structure of the yarn. When the yarn is stretched and breaks, the nanofibers do not play a role. Electron microscope images show that the embedding of nanofibers does not alter the yarn’s primary structure. Instead, the nanofibers attach to the surrounding traditional fibers in single or multiple strands, filling the pores of the short-fiber yarn. As shown in Figure 3b, the number of hairiness roots larger than 3 mm was similar in both yarns, which was about 31 roots/10 cm, and a large number of PAN nanofibers were embedded in the yarn body of EN yarn. Intuitively, it is thought that the embedding of a large number of fine nanofibers would cause the hairiness index of the yarns to be too high, but the hairiness indices of the two yarns were similar. This is because polyacrylonitrile is a polar polymer; its surface will accumulate a large number of charges from contact with aramid and flame-retardant viscose fibers during spinning, and the short fibers and nanofibers will produce electrostatic adsorption such that more nanofibers are in the form affixed to the short fibers, as shown in Figure 2; hence, the nanofibers have less impact on the yarn hairiness coefficient. Knitted fabrics were prepared using the two yarns and the basic breaking strengths of the knitted fabrics were measured. As shown in Figure 3c, the breaking strength of traditional fabrics is 370.4 N, while that of inlaid fabrics is 374.43 N. The breaking strength of the two types of fabrics is similar, mainly because the yarn strength of the fabrics is similar and the two fabrics have the same structure, so there is no significant difference in the strength of the two fabrics. As shown in Figure 3d, the pilling performance of the two knitted fabrics is poor; both fabrics exhibited a weight loss rate exceeding 1.6% after 99 friction cycles, receiving a subjective rating of Level 3. which is mainly due to the fact that the knitted fabrics were formed by the loops stringing each other and thus, the structure was more lax, and the fibers were more prone to move and form lint in friction, eventually entangling into a ball. The pilling performance of EF fabrics did not decrease significantly, which is due to the low content of nanofibers in the fabrics themselves, so the weight loss rate of the fabrics were almost unchanged. The shorter length and finer diameter of the nanofibers result in little or no formation of the traditional fabric pilling pattern. As shown in Figure 3e, the bending stiffness of the two fabrics is similar, which is about 23 mN·cm, and the results prove that the embedded nanofibers do not affect the softness of the yarn. Although the embedded yarn contains a large number of nanofibers, the diameter of nanofibers is much smaller, which does not affect the main body of the staple yarn significantly, and the nanofibers do not exist in the yarn in a continuous form, but in micron over millimeter lengths, so the fabrics do not affect their cross-sectional moments of inertia when bending. As shown in Figure 3f, the air permeability of the two fabrics is similar, which is about 1190 mm/s. The main reason for the similarity of the air permeability of the two fabrics is that they are largely influenced by fabric organization. The main reason for the similar permeability of the two fabrics is that the biggest influence factor of the fabric permeability, for knitted fabrics, is the fabric’s organizational structure, which itself has a large pore space. Although the nanofibers fill the pore space of the yarn and decrease the air permeability of the yarn, in the face of a larger fabric pore space, the impact of this point is negligible.

### 3.2. Fabric Moisture Conductivity

**Figure 4:**
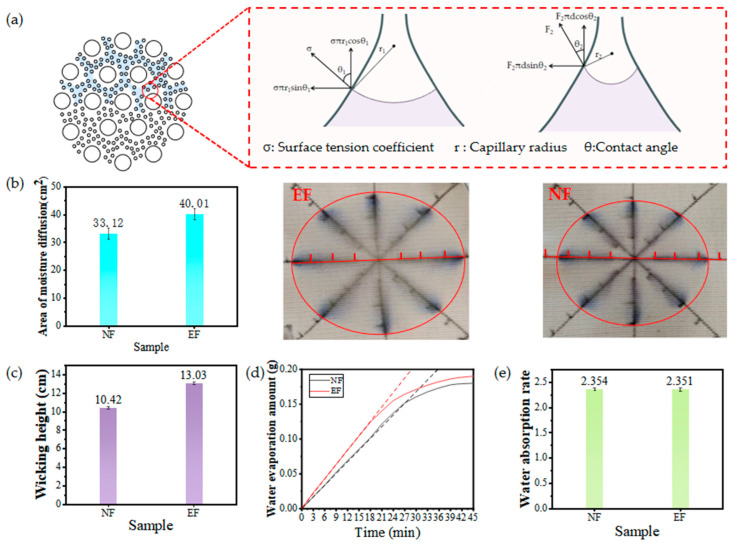
Moisture conductivity of fabrics: (**a**) Schematic of moisture conductivity of fabrics; (**b**) vertical core absorption height of fabrics; (**c**) drip diffusion area of fabrics; (**d**) moisture evaporation rate of fabrics; (**e**) moisture absorption rate of fabrics.

As shown in Figure 4a, the ideal cross-sectional geometric model of EY features a large number of uniformly dispersed nanofibers distributed throughout the yarn, forming capillary channels that extend throughout the entire yarn structure. According to the Young–Laplace equation (Equation (2)) and its modified formula (Equation (3)), it is known that capillary pressure is inversely proportional to capillary radius. The capillary channels formed between nanofibers and nanofilaments have smaller diameters, resulting in stronger capillary forces. Additionally, the capillary tubes formed between traditional fibers and nanofibers randomly generate differential capillary effects, further promoting rapid water flow [37]. Additionally, an interface forms around traditional fibers where they come into contact with nanofibers, and this differential capillary effect accelerates water migration. When liquid water enters the embedded structure composite yarn, under the combined action of hydrostatic pressure and capillary pressure, water rapidly penetrates into the yarn body. Once inside the yarn body, under the influence of differential capillary pressure, water aggregates and is transported within the capillary tubes formed by nanofibers.

(2) $$P=\frac{2\gamma \cos\theta }{r}$$

(3) $$\Delta P=2\gamma \cos\theta \left(\frac{1}{r_{1}}\right.-\left.\frac{1}{r_{2}}\right)$$

The purpose of measuring indicators such as the water droplet spread area and vertical capillary rise height of the fabrics is to evaluate their vertical moisture transfer performance, which is directly related to the fabric’s moisture absorption, water absorption, quick-drying properties, and comfort. As shown in Figure 4b, the fabric was cut into 10 × 10 cm^2^, with a coordinate grid marked at 1 cm intervals on the fabric surface. A 200 μL water droplet was placed at the center of the grid. Due to the fabric’s structural characteristics, the moisture diffusion area exhibited an approximate elliptical shape, prompting the use of the ellipse area calculation method. Experimental data indicate that the water diffusion area for EF fabric is 40.01 cm^2^, while that for NF fabric is 33.12 cm^2^. This demonstrates that the presence of nanofibers within the yarn structure increases the water diffusion area of EF fabric by 20% compared with NF fabric. The nanofibers form finer capillary channels between themselves, generating stronger capillary forces. Water is subjected to more potent capillary forces within the yarn, enabling spontaneous and rapid conduction. Concurrently, influenced by the knit fabric structure, water more readily propagates along the yarn’s axial direction, resulting in an elliptical shape for the water spread area. As shown in Figure 4c, the vertical core absorption height of EF fabric was 13.03 cm/30 min and that of NF fabric was 10.42 cm/30 min, and the vertical core absorption performance of EF fabric was 25% higher than that of NF fabric. The fabric core absorption height is mainly determined by the radius of the capillary channels of the yarns in the fabric, the fabric structure, and the hydrophilicity of the fibers. The fiber types of the two fabrics are the same, and the two fabric structures are the same, so the higher core absorption height of EF fabric is only due to the embedding of a large number of nanofibers in the yarn, which constitutes a finer capillary channel to generate a larger capillary force, resulting in a higher transfer of water in the vertical direction. As shown in Figure 4d, the fabric’s moisture evaporation rate exhibits rapid evaporation during the first 20 min due to the swift evaporation of surface moisture. Subsequently, the evaporation rate gradually slows as moisture from within the fabric slowly and progressively migrates to the surface for evaporation. This process is slow, so we compare the slope of the moisture evaporation rate curve during the first 20 min as the fabric’s moisture evaporation rate. The moisture evaporation rate of EF fabric is 0.007 g/min, while that of NF fabric is only 0.0052 g/min. The moisture evaporation rate of EF fabric is nearly 35% higher than that of NF fabric. The moisture diffusion area of EF fabric is larger than that of NF fabric, leading to its higher evaporation rate. Concurrently, the macromolecular chains of flame-retardant viscose fibers contain abundant hydrophilic groups. These hydrophilic groups, along with water molecules bound by chemical bonds, “firmly lock” moisture around the flame-retardant viscose fibers. Meanwhile, nanofibers form moisture transport channels, reducing contact between the flame-retardant viscose fibers and moisture, thereby further enhancing the moisture diffusion rate. As shown in Figure 4e, the water absorption rates of the two fabrics are essentially comparable. Although EF fabric exhibits stronger capillary forces toward water, once immersed, water saturates the entire fabric, meaning capillary action does not affect its water content. The nanofibers embedded within the yarn structure demonstrate poor water absorption properties and are present in low concentrations, thus failing to influence the fabric’s overall water absorption capacity. Consequently, the incorporation of nanofibers has a negligible impact on the fabric’s water absorption rate.

### 3.3. Fabric Antimicrobial Properties

**Figure 5:**
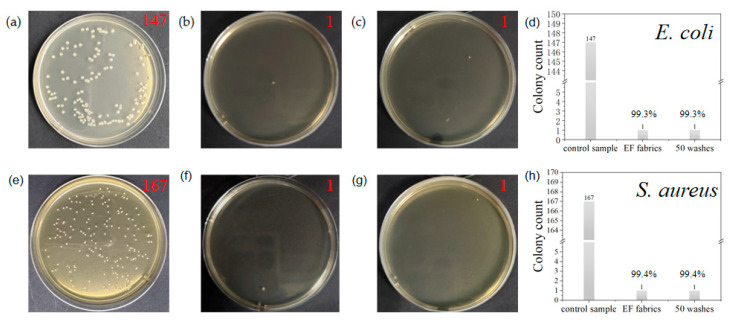
Comparison of fabric antibacterial performance: (**a**) Colony count of *E. coli* control group; (**b**) colony count of EF fabric after *E. coli* antibacterial testing; (**c**) colony count of EF fabric after antibacterial testing following 50 washes; (**d**) EF fabric bacterial inhibition rate data chart for *E. coli*; (**e**) colony count of control fabric after antibacterial testing against *S. aureus*; (**f**) colony count of EF fabric after antibacterial testing against *S. aureus*; (**g**) colony count of EF fabric after antibacterial testing against *S. aureus* following 50 washes; (**h**) EF fabric bacterial inhibition rate data chart for *S. aureus*.

TR has been widely demonstrated to possess antimicrobial properties. Its growth inhibitory properties are mediated by blocking lipid synthesis through the specific inhibition of Nadh-dependent enoyl acyl carrier protein (ACP) reductase or inhibition of enoyl reductase. The TR released from nanofibers resulted in bacterial inactivation, and *E. coli* and *S. aureus* were selected as model bacteria in this study. As shown in Figure 5a, the control sample of *E. coli* after 10,000 dilutions contained 147 colonies. As shown in Figure 5b, after subsequent antibacterial testing of EF fabric against *Escherichia coli*, only one colony was observed on the plate. As shown in Figure 5c, after undergoing 50 water-wash treatments, EF fabric exhibited only a single colony in the *E. coli* resistance test. As shown in Figure 5d, EF fabric achieved a 99.3% inhibition rate against *E. coli* bacteria. Notably, even after 50 wash cycles, EF fabric maintained a 99.3% inhibition rate against *E. coli*, demonstrating exceptional antibacterial performance against this pathogen. Furthermore, the nanofiber yarn exhibited outstanding durability, showing no degradation even when subjected to intense external impact forces. As shown in Figure 5e, the control sample of *Staphylococcus aureus* after 10,000 dilutions contained 167 colonies. As shown in Figure 5f, after the subsequent antibacterial test against *Staphylococcus aureus* on EF fabric, only one colony was observed on the plate. As shown in Figure 5g, after 50 washes, EF fabric exhibited only a single colony on the agar plate when tested for *Staphylococcus aureus*. As shown in Figure 5h, EF fabric demonstrated an inhibition rate of up to 99.4% against *Staphylococcus aureus*. Notably, even after 50 wash cycles, the fabric maintained its 99.4% antibacterial efficacy, exhibiting exceptional antimicrobial performance against this pathogen. The release process of *TR* in fabrics can be divided into three stages. In the first stage (≤50 h), the drug release was rapid, which was due to the rapid release of TR directly from the surface of the nanofibers in the fabric. In addition, the increase in TR content resulted in more of the drug on the surface of the nanofibers, causing a burst release in the initial phase. The second phase lasted longer (50–480 h) with continuous release of TR into PBS. In the final stage (480 h), it takes a long time for the TR within the nanofibers to be released due to the non-degradable nature of PAN nanofibers. When *TR* is released from EF fabrics, there are three diffusion pathways for the drug: diffusion from the surface of the nanofibers, diffusion through the interfiber space, and diffusion through the fiber “wall”. In the second stage of longer duration, *TR* has two main diffusion paths, one part diffuses through the interfiber space, and the other part diffuses through the fiber “wall”, which forms an additional barrier and prolongs the diffusion paths and diffusion time, indicating that the antimicrobial performance of EF fabrics against *Escherichia coli* and *Staphylococcus aureus* is not only excellent but also that the antimicrobial function is released for a long time with high functional reliability. This point has been explored in the previous work [38].

## 4. Conclusions

This study reports a functionally integrated knitted firefighting fabric prepared from aramid 1313/flame-retardant viscose fiber/35/65 blended yarn, which is both moisture-conducting and antimicrobial. The innovative process embeds nanofibers with antimicrobial properties into the conventional yarn body: the antimicrobial properties possessed by the nanofibers confer excellent antimicrobial properties to the yarn, while the embedding of the nanofibers alters the structure of the conventional yarn body to achieve efficient moisture conductivity. The experimental results show that the functional integrated fabric prepared using this embedded yarn maintains an antibacterial effect of over 99% against *E. coli* and *S. aureus* after 50 washes, with significantly improved moisture-transporting performance. It should be noted that this manufacturing process inevitably involves the use of DMF, which may have certain environmental impacts.

## Data Availability

The original contributions presented in this study are included in the article. Further inquiries can be directed to the corresponding authors.
